# Population Genomics of *Francisella tularensis* subsp. *holarctica* and its Implication on the Eco-Epidemiology of Tularemia in Switzerland

**DOI:** 10.3389/fcimb.2018.00089

**Published:** 2018-03-22

**Authors:** Matthias Wittwer, Ekkehard Altpeter, Paola Pilo, Sebastian M. Gygli, Christian Beuret, Frederic Foucault, Rahel Ackermann-Gäumann, Urs Karrer, Daniela Jacob, Roland Grunow, Nadia Schürch

**Affiliations:** ^1^Spiez Laboratory, Federal Office for Civil Protection, Spiez, Switzerland; ^2^Swiss National Reference Center for Francisella tularensis (NANT), Spiez, Switzerland; ^3^Swiss Federal Office of Public Health, Bern, Switzerland; ^4^Department of Infectious Diseases and Pathobiology, Institute of Veterinary Bacteriology, University of Berne, Berne, Switzerland; ^5^Swiss Tropical and Public Health Institute, Basel, Switzerland; ^6^University of Basel, Basel, Switzerland; ^7^Mabritec AG, Riehen, Switzerland; ^8^Swiss National Reference Centre for Tick-Transmitted Diseases (NRZK), Spiez, Switzerland; ^9^Cantonal Hospital Winterthur, Winterthur, Switzerland; ^10^ZBS 2, Center for Biological Threats and Special Pathogens, Robert Koch Institute, Berlin, Germany

**Keywords:** tularemia, whole genome sequencing (WGS), ticks, *Francisella tularensis* subsp. holarctica, ecology, epidemiology of infectious diseases, phylogenomics, canSNPs

## Abstract

Whole genome sequencing (WGS) methods provide new possibilities in the field of molecular epidemiology. This is particularly true for monomorphic organisms where the discriminatory power of traditional methods (e.g., restriction enzyme length polymorphism typing, multi locus sequence typing etc.) is inadequate to elucidate complex disease transmission patterns, as well as resolving the phylogeny at high resolution on a micro-geographic scale. In this study, we present insights into the population structure of *Francisella tularensis* subsp. *holarctica*, the causative agent of tularemia in Switzerland. A total of 59 *Fth* isolates were obtained from castor bean ticks (*Ixodes ricinus)*, animals and humans and a high resolution phylogeny was inferred using WGS methods. The majority of the *Fth* population in Switzerland belongs to the west European B.11 clade and shows an extraordinary genetic diversity underlining the old evolutionary history of the pathogen in the alpine region. Moreover, a new B.11 subclade was identified which was not described so far. The combined analysis of the epidemiological data of human tularemia cases with the whole genome sequences of the 59 isolates provide evidence that ticks play a pivotal role in transmitting *Fth* to humans and other vertebrates in Switzerland. This is further underlined by the correlation of disease risk estimates with climatic and ecological factors influencing the survival of ticks.

## Introduction

Classification of organisms according to inherent characteristics of their genome has become an indispensable principle in molecular biology. Over the years, progress in sequencing technologies led to an explosion in the amount of available sequencing data and an ever-increasing taxonomic resolution. This development culminated in the advent of massive parallel sequencing methods that allow the classification and characterisation of an organism on a whole genome scale, opening new perspectives in molecular forensics, ecology, and epidemiology. The benefits of whole genome sequencing (WGS)-based classification are most pronounced when applied to genetically monomorphic organisms, for example *Mycobacterium tuberculosis* (Lee et al., 2015; Stucki et al., 2016), *Bacillus anthracis* (Girault et al., 2014) and *Francisella tularensis* (Dwibedi et al., 2016).

Tularaemia is caused by the gram negative, facultative intracellular bacterium *F. tularensis*. Due to its very low infective dose and high mortality when inhaled as aerosol, the organism is listed as a category A biothreat agent (Rotz et al., 2002). Two subspecies are responsible for most of the tularemia cases. *F. tularensis* subsp. *tularensis* (*Ftt*), and *F. tularensis* subsp. *holarctica* (*Fth*). In Europe, the less virulent *Fth* occurs and generally causes sporadic cases, of which the first were documented in the 1930s (Jusatz, 1961). *Fth*, however, occurs throughout the northern hemisphere. Since the late 1990s, case numbers are increasing and outbreaks exceeding 1,000 cases were reported in the Czech Republic, Hungary, Spain (Pérez-Castrillón et al., 2001), Sweden and Finland (Maurin and Gyuranecz, 2016). It is thought that in the United States the majority of *F. tularensis* infections are due to transmission from tick bites (Petersen et al., 2009) whereas in Europe, the situation is less clear. In Scandinavia, *Fth* is transmitted predominantly by mosquitoes and it is believed that the pathogen persists in an aquatic life cycle with mosquitoes, mosquito larvae and rodents (Desvars et al., 2015). The terrestrial lifecycle, with arthropods as reservoirs and small terrestrial rodents and lagomorphs, as susceptible hosts, is predominant in most European countries including Switzerland. In these countries, direct contact with infected rodents and lagomorphs seems to be the main route of infection. Concerning the transmission through arthropod vectors, the estimated percentage of tularaemia cases due to tick bites varies between 12% (Slovakia) and 26% (France) (Maurin and Gyuranecz, 2016). Since tularaemia is a notifiable disease in Switzerland, epidemiological data is collected from patient reports sent from the initial point of care to the health authorities. According to this information, tick bites could be associated with 47% of the tularaemia cases reported during the last 10 years. However, the quality of questionnaire-based data is often limited. Additionally, the confirmation of a causal connection between arthropod bites and tularaemia cases using a method providing adequate discriminative power has been lacking. In this study the epidemiology, routes of transmission, and phylo-geographic properties of tularaemia in Switzerland are delineated based on 59 sequenced genomes of *Fth* isolated from humans, animals and ticks (Table 1).

**Table 1:** Overview of the 59 whole genome sequenced *Fth* strains from Switzerland used in this study.

**Sample ID**	**Alternative ID**	**Source, year**	**Clade**	**Sequencing institute/Sample provider**	**Length of assembly**	**Contigs**	**N50**	**Accession number**	
FT 29	JF5340^a^	Human, 2012	B.33	SL/IVB	1798068	102	26695	SAMN08108654	PKBF00000000
FT 31	JF5370^a^	Human, 2012	B.33	SL/IVB, ADMED	1794649	100	27062	SAMN08108656	PKBD00000000
FT 65	JF5405^a^	Hare, 2012	B.33	SL/IVB	1802560	102	27621	SAMN08108682	PKAE00000000
FT 66	JF5468^a^	Hare, 2013	B.33	SL/IVB	1823931	98	27394	SAMN08108683	PKAD00000000
FT 70	JF5609	Monkey, 2014	B.33	SL/IVB	1823454	96	27316	SAMN08108687	PJZZ00000000
FT 22	JF4456^a^	Human, 2008	B.45	SL/IVB	1801181	95	27456	SAMN08108648	PKBL00000000
FT 32^b^		Human, 2012	B.45	SL/IVB	1812310	95	27658	SAMN03774932	PKAX00000000
FT 41	FT6_D12	Tick, 2012	B.45	SL	1782791	96	26944	SAMN08108662	PKAK00000000
FT 56	JF5353^a^	Marten, 2012	B.45	SL/IVB	1818006	97	27313	SAMN08108675	PKAC00000000
FT 67	JF5487	Hare, 2013	B.45	SL/IVB	1822695	96	27391	SAMN08108684	PJZY00000000
FT 71	JF5611	Hare, 2014	B.45	SL/IVB	1812653	98	27006	SAMN08108688	PKBP00000000
FT 14	JF3829	Lion Tamarin, 2004	B.46	SL/IVB	1801041	95	27712	SAMN08108642	PKBR00000000
FT 17	JF4128/FDC304^b^	Human, 2008	B.46	SL/IVB	1778390	101	24282	SAMN08108644	PKBH00000000
FT 27	JF5141	Human, 2011	B.46	SL/IVB	1807038	109	26708	SAMN08108652	PKBG00000000
FT 28	JF5338	Human, 2012	B.46	SL/IVB	1788115	102	26847	SAMN08108653	PKBT00000000
FT 11	JF3821	Hare, 1997	B.49	SL/IVB	1801451	95	27456	SAMN08108640	PKBQ00000000
FT 16	JF4092	Hare	B.49	SL/IVB	1801566	95	27456	SAMN08108643	PKAQ00000000
FT 50		Human 2014	B.49	SL	1823626	96	27535	SAMN08108669	PKBO00000000
FT 18	JF4212/FDC305^b^	Human, 2008	B.53	SL/IVB	1801159	95	27712	SAMN03773856	PKBB00000000
FT 36		Human, 2014	B.53	SL	1817604	96	27313	SAMN08108658	PKAS00000000
FT 48		Human, 2014	B.53	SL	1817319	96	27313	SAMN08108667	PKAJ00000000
FT 57	JF5369^a^	Hare, 2012	B.53	SL/IVB	1809350	97	27005	SAMN08108676	PKAW00000000
FT 38	FT9C_G7^b^	Tick, 2012	B.59	SL	1811355	96	27676	SAMN03774936	PKAU00000000
FT 42	FT11_B4	Tick, 2012	B.59	SL	1783150	96	26974	SAMN08108663	PKBI00000000
FT 45	FT8_F4	Tick, 2012	B.59	SL	1814235	96	27658	SAMN03774935	PKAO00000000
FT 46	FT8_B3	Tick, 2012	B.59	SL	1780077	95	26974	SAMN08108665	PKAN00000000
FT 26	JF5142	Human, 2011	B.61	SL/IVB	1800278	102	27104	SAMN08108651	PKAM00000000
FT 52	FT21_C4	Tick, 2013	B.61	SL	1801053	95	27456	SAMN08108671	PKAH00000000
FT 53	FT22_F9	Tick, 2013	B.61	SL	1801183	95	27456	SAMN08108672	PKBA00000000
FT 54	FT22_H4	Tick, 2013	B.61	SL	1801200	95	27456	SAMN08108673	PKAZ00000000
FT 59	JF5375^a^	Hare, 2012	B.61	SL/IVB	1819651	97	27313	SAMN08108678	PKAY00000000
FT 37	FT14_F1	Tick, 2012	B.62	SL	1779923	96	26888	SAMN08108659	PKAV00000000
FT 39	FT14_C4	Tick, 2012	B.62	SL	1780016	96	26888	SAMN08108660	PKAT00000000
FT 40	FT17_C9	Tick, 2012	B.62	SL	1782242	96	26888	SAMN08108661	PJZW00000000
FT 43	FT16_B1^b^	Tick, 2012	B.62	SL	1805387	95	27658	SAMN03774942	PKBU00000000
FT 44	FT16_E6	Tick, 2012	B.62	SL	1795991	96	27077	SAMN08108664	PKBV00000000
FT 47	FT16_G8^b^	Tick, 2012	B.62	SL	1781679	96	26888	SAMN08108666	PKBS00000000
FT 75		Human, 2015	B.62	SL	1797368	95	27019	SAMN08108690	PKBM00000000
FT 08		Human, 2003	B.85	SL/IVB	1775380	133	21731	SAMN08108638	PKAG00000000
FT 10	JF3820^a^	Hare, 1998	B.85	SL/IVB	1772027	102	23187	SAMN08108639	PKAB00000000
FT 13	JF3826^a^	Marmoset, 1996	B.85	SL/IVB	1801174	95	27456	SAMN08108641	PKBK00000000
FT 20	JF4429/FDC310^b^	Human, 2008	B.85	SL/IVB	1814653	96	27257	SAMN03773858	PKAP00000000
FT 60	JF5380^a^	Hare, 2012	B.85	SL/IVB	1801233	95	27456	SAMN08108679	PKAL00000000
FT 68	JF5525	Hare, 2014	B.85	SL/IVB	1818684	96	27313	SAMN08108685	PKBY00000000
FT 24	JF5002	Human, 2011	B.87	SL/IVB	1786520	97	26974	SAMN08108649	PKBW00000000
FT 51		Human, 2014	B.87	SL	1820050	96	27535	SAMN08108670	PKBJ00000000
FT 55	JF5345	Mouse, 2012	B.87	SL/IVB	1825678	100	27313	SAMN08108674	PKBE00000000
FT 64	JF5410^a^	Mouse, 2012	B.87	SL/IVB	1819162	96	27313	SAMN08108681	PJZX00000000
FT 07		Human, 2008	B.88	SL	1782483	96	26925	SAMN08108637	PKBN00000000
FT 25	JF5048	Human, 2011	B.88	SL/IVB	1797415	97	27104	SAMN08108650	PKBC00000000
FT 30	JF53461	Human, 2012	B.88	SL/IVB	1783341	96	27105	SAMN08108655	PKBX00000000
FT 19	JF4242/FDC306^b^	Hare	B.89	SL/IVB	1801171	95	27712	SAMN03773857	PKAI00000000
FT 33		Human, 2013	B.89	SL	1820901	96	27535	SAMN08108657	PKAF00000000
FT 73		Human, 2015	B.89	SL	1797389	95	27712	SAMN08108689	PKAR00000000
FT 05		Human, 2008	B.90	SL/IVB	1797365	95	27456	SAMN08108636	PKAA00000000
FT 58	JF5374^a^	Hare, 2014	B.90	SL/IVB	1819228	96	27313	SAMN08108677	MQVE00000000
FT 63	JF5393^a^	Hare, 2012	B.90	SL/IVB	1824976	96	27435	SAMN08108680	MQVC00000000
FT 49		Human, 2014	B.91	SL	1822738	96	27535	SAMN08108668	MQVF00000000
FT 69	JF5597	Hare, 2014	B.91	SL/IVB	1818326	96	27313	SAMN08108686	MQVD00000000

*SL, Spiez laboratory, Spiez, Switzerland; IVB, Institute of Veterinary Bacteriology, Bern, Switzerland; ADMED, Analyses et Diagnostics Medicaux, La Chaux de Fonds, Switzerland*.

a *Origgi et al. (2014)*.

b *Dwibedi et al. (2016)*.

## Materials and methods

### Collection of ticks

Between 2009 and 2015 a total of 120,000 questing ticks were collected at 165 collection sites throughout Switzerland by flagging low vegetation. Ticks were randomly identified based on morphological characteristics (Keirans, 1997) and immediately stored at −80°C. Subsequently, ticks were washed once in 75% ethanol and twice in deionized water, dried on paper towels, and sorted into pools of 10 nymphs or 5 adult male or female *I. ricinus* ticks. Pooled samples were stored at −80°C until further processing (Gäumann et al., 2010).

### Homogenization of ticks

Tick homogenates were prepared from frozen tick pools of 10 nymphs or 5 adult female or male ticks as follows: 600 μl of cold PBS was added to each frozen tick pool. Samples were immediately homogenized using the TissueLyser system (Qiagen). Briefly, one 3-mm tungsten carbide bead (Qiagen) was added to each tube (collection microtubes; Qiagen), and tick pools were homogenized for 4 min at 30 Hz. After centrifuging the samples for 5 s at 3,220 g, supernatants were collected and split for further use: One 200 μl aliquot was used for DNA extraction and four 150 μl aliquots were immediately frozen in liquid nitrogen and stored at −80°C. To increase success rates of subsequent cultivation efforts, 40 μl glycerol (100%) was added as a cryoprotectant to two aliquots.

### Screening of tick homogenates for the presence of *Fth*

DNA was automatically extracted using a magnetic bead based protocol (QiaSymphony, QIAGEN). In total, 16,000 tick pools were screened for the presence of *Fth* using a one-step real-time RT-PCR assay based on the VNTR marker Ft-M19 (Byström et al., 2005). The Ft-M19 PCR positive samples were confirmed by an in-house real-time RT-PCR assay targeting the *fopA* gene (Table 2).

**Table 2:** PCR assays targeting the *Francisella* Outer Membrane Protein A used for *F. tularensis* ssp. screening.

**Target**		**Sequence**	**Primer length**
FopA-F	Forward primer	5′-CAAATCTAGCAGGTCAAGCAACAG-3′	24
FopA-R	Reverse primer	5′-CACTTGCTTGAACATTTCTAGATAGTTCA-3′	29
FopA-S	Probe	FAM-5′-TGCTTGGGATGTGGGTGGTGGTC-3′-BHQ1	23

*Amplicon length = 270 bp*.

### Isolation of *Fth* from homogenized tick samples

Aliquots of the PCR positive samples were cultivated. Briefly, frozen aliquots were quickly thawed and 50 μl each was inoculated in 10 ml Broth Medium T (Becker et al., 2016), as well as streaked on Chocolate Agar PolyViteX (BioMérieux) and Thayer Martin VCNT Neisseria Selective Agar (Oxoid). Plates and liquid cultures were incubated at 37°C using GENBox C02 (BioMérieux) for 2–6 days. Presumptive *Fth* cultures were confirmed by real-time PCR as described above (fopA; Table 2).

### DNA isolation for WGS

All manipulations with live cultures were performed in a BSL 3 containment laboratory (approval number: A110502/3). Isolates of *Fth* were grown on Chocolate Agar PolyViteX (BioMérieux) for 2 days and colonies were harvested and suspended in 500 μl AVL Buffer (Qiagen). Lysates were heat inactivated for 15 min at 100°C and DNA was subsequently extracted using the EZ1 or the EZ1 Advanced robot (Qiagen) according to the manufacturer's instructions (EZ1, Tissue Kit, Bacteria Card, Qiagen).

### Human samples

In its function as the Swiss national reference center for tularemia in humans, the Spiez Laboratory maintains a culture collection of *Fth* isolates derived from routine clinical diagnostics. The patient and epidemiological data are collected by the Federal Office of Public Health according to the Epidemics Law and Ordinance (SR 818.101.126).

### Animal samples

Strains isolated from animals were selected from the strain collection of the Institute of Veterinary Bacteriology, Vetsuisse Bern, Switzerland, which is the national reference center for tularemia in animals.

### Whole genome sequencing

The 59 *Fth* isolates were sequenced on the Illumina HiSeq 2500 platform using the TruSeq paired end chemistry for library preparation. The average read length was 126 bp and the average insert size was 350 bp (standard deviation = 87.593, lower quantile = 272, upper quantile = 488). Prior to the assembly the ~50,000,000 sequencing reads were quality trimmed with *Trimmomatic V0.32* (Bolger et al., 2014).

The quality trimmed reads were assembled using *SPAdes*-3.10.1 (Bankevich et al., 2012) resulting in ~100 contigs with an average N50 of 27,000 bp. The coverage of the *Fth* genomes was >1,000 × in all samples. In order to use the pan genome pipeline *Roary* (Page et al., 2015), gff3 annotation files were generated with Prokka (Seemann, 2014) using the Franco-Iberian *Fth* FTNF002-00 (NC_009749) strain as reference. Based on these gff3 files, the core- and pan-genomes were inferred using *Roary* by applying a 95% *blastp* identity threshold. A given gene had to be present in 100% of the samples to be included into the core-genome. *Mafft* (Katoh et al., 2002) was used for core genome alignment. Ambiguously aligned regions and Indels were removed from the *Mafft* alignment by *Gblocks* (Castresana, 2000) and *SEQfire* (Ajawatanawong et al., 2012). Maximum likelihood phylogenies were inferred with *PhyML* (Initial tree: BioNJ; Tree topology search: NNIs; Model of nucleotide substitution: TN93; Bootstrap replicates: 1000) (Guindon et al., 2010). Mutations in the sequenced strains relative to the reference genomes were identified with *breseq* (Deatherage and Barrick, 2014).

To designate the sequenced strains to the established canSNP node nomenclature the software canSNPer (Lärkeryd et al., 2014) was used. Naming of the novel subtypes found in this study is in agreement with the canSNP nomenclature scheme assigning an increasing ordinal number to each newly identified SNP. To implement the novel subtypes in the canSNPer tool, the text files containing the SNP positions and the tree topology were adjusted accordingly.

Calculation of average nucleotide identity using standard MUMmer algorithm (ANIm) from whole genome was done using a python program pyani (v0.2.4). Hierarchical cluster analysis of ANI correlation matrix was done using package hclust (R v3.4.3).

### Spatial statistics

Phylogeographic interpolation was performed with the R package Phylin (Tarroso et al., 2015). Calculation of spatial disease clusters was performed (carried out) with the R package DCluster (Hornik et al., 2003).

## Results

### Prevalence of *Fth* in Swiss ticks

A total of 120,000 questing ticks were analyzed and only 25 tick homogenates were positive for *Fth* by PCR. This corresponds to a prevalence of ~0.02%.

Fourteen *Fth* isolates were successfully recovered from the 25 positive tick homogenates. The recovery from glycerol preserved samples was slightly improved and the isolation was often easier from Neisseria Agar than from Chocolate Agar due to reduced contaminating flora.

### Phylogenetic context of Swiss *Fth* isolates

ANIm is a standard *in silico* method for bacterial species delimitation with a threshold value of 94–96%. Here, ANIm was applied to validate that 59 isolates belong to *F. tularensis* subsp*. holarctica*. Whole genomes were compared to type strains: *F. tularensis* subsp*. tularensis* NIH B-38, *F. tularensis* subsp. *holarctica* FTNF002-00, *F. tularensis* subsp*. mediasiatica* FSC147, and *F. tularensis* subsp. *novicida* U112. In this small dataset, we found that an ANIm threshold of 99.5% could distinguish subspecies of *F. tularensis*. All 59 isolates showed >99.9% ANI identity to *F. tularensis* subsp*. holarctica* FTNF002-00 and *F. tularensis* subsp*. holarctica* FSC200.

**Figure 1:**
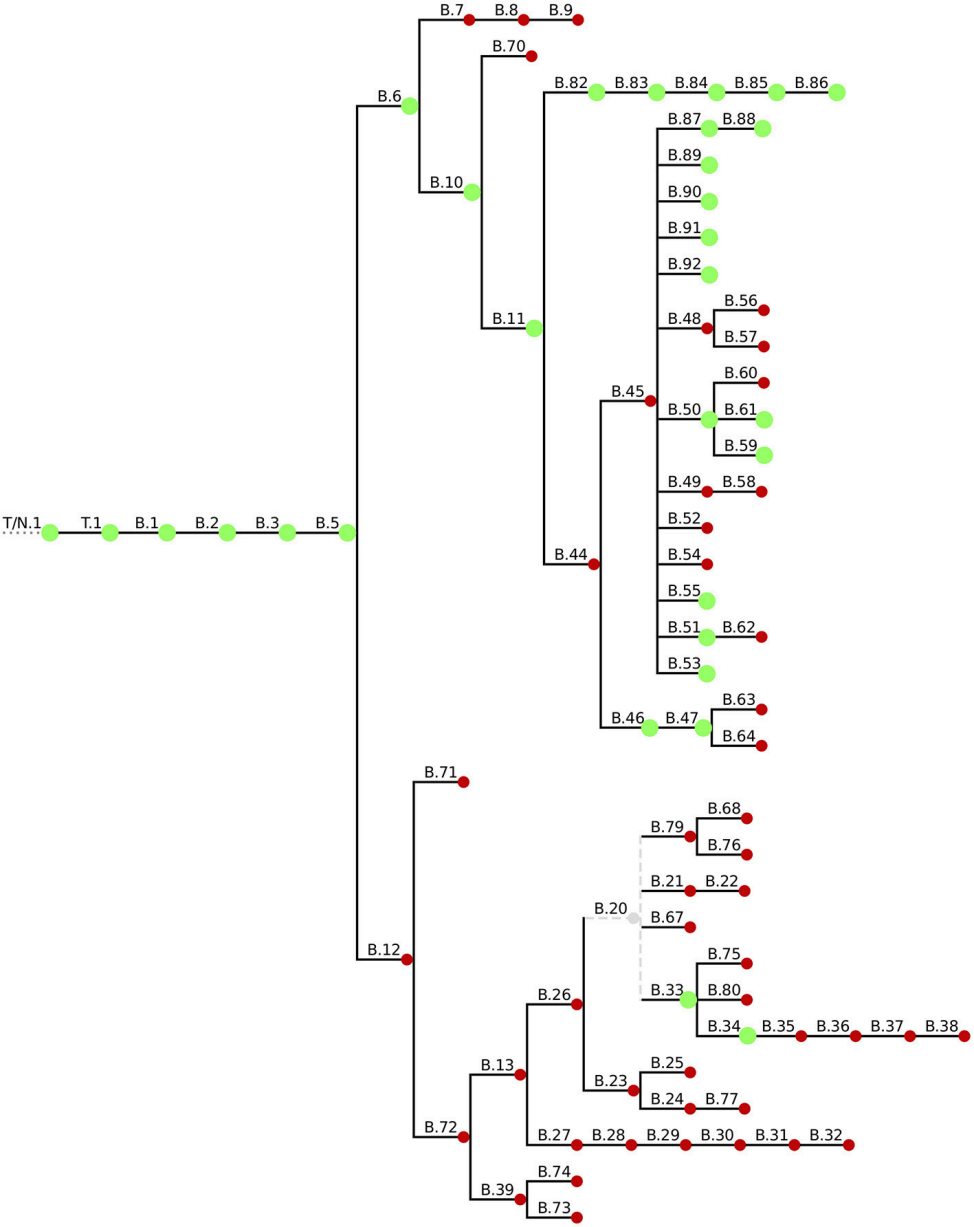
Overview of the current canSNP typing scheme. Green blobs indicate the canSNP types found in the 59 sequenced Swiss *Fth* isolates.

To put the 59 Swiss *Fth* whole genome sequences in a global phylogenetic context we used a canonical SNP (canSNP) approach (Lärkeryd et al., 2014). According to this typing method, 54 strains (91.5%) were assigned to the B.11 clade, which is predominant in Western Europe including Switzerland (Pilo et al., 2009; Vogler et al., 2009; Dwibedi et al., 2016). Three animal and two human isolates (11%) belong to the Northeast European B.12 clade (Figure 1).

**Figure 2:**
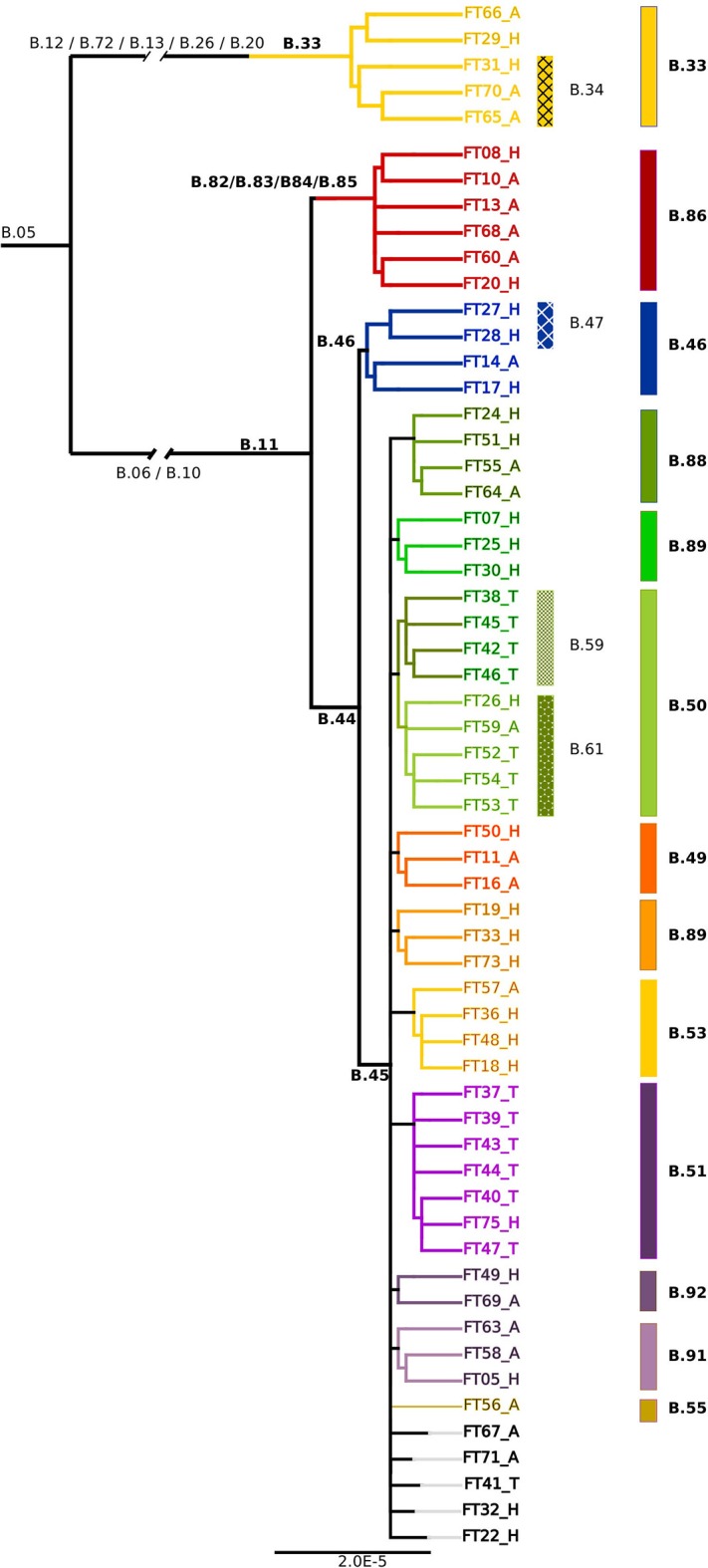
Phylogenetic relationships based on the core genome alignment of 59 sequenced *Fth* strains. The clades and subclades are labeled according to the canSNP nomenclature. The ending letters of the tip labels indicate the source of the isolates (H, Human; A, Animal; T, Tick).

To increase the phylogenetic resolution a core genome SNP analysis based on a 1477202bp alignment was applied and revealed a similar branching pattern of the B.11 clade as described in the European survey by Dwibedi et al. (2016). Clade B.45 was represented by 44 (18 human, 14 tick, 12 animal) and B.46 by four isolates (three human, 1 animal). In addition, six isolates (2 human, 4 animal) were assigned to a clade, which was previously represented by one Swiss isolate only. This novel B.11 subclade is distinguished by 5 SNPs (B.82/B.83/B.84/B.85/B.86; Figure 2).

Except of the clades B.48 and B.52 exclusively found in Spain, as well as the French B.54 clade, all subclades derived from B.45 were represented among the 59 sequenced isolates. In addition, five novel subclades deriving from B.45 could be identified (B.87/B.88, B.89, B.90, B.91, B.92; Figure 2).

**Figure 3:**
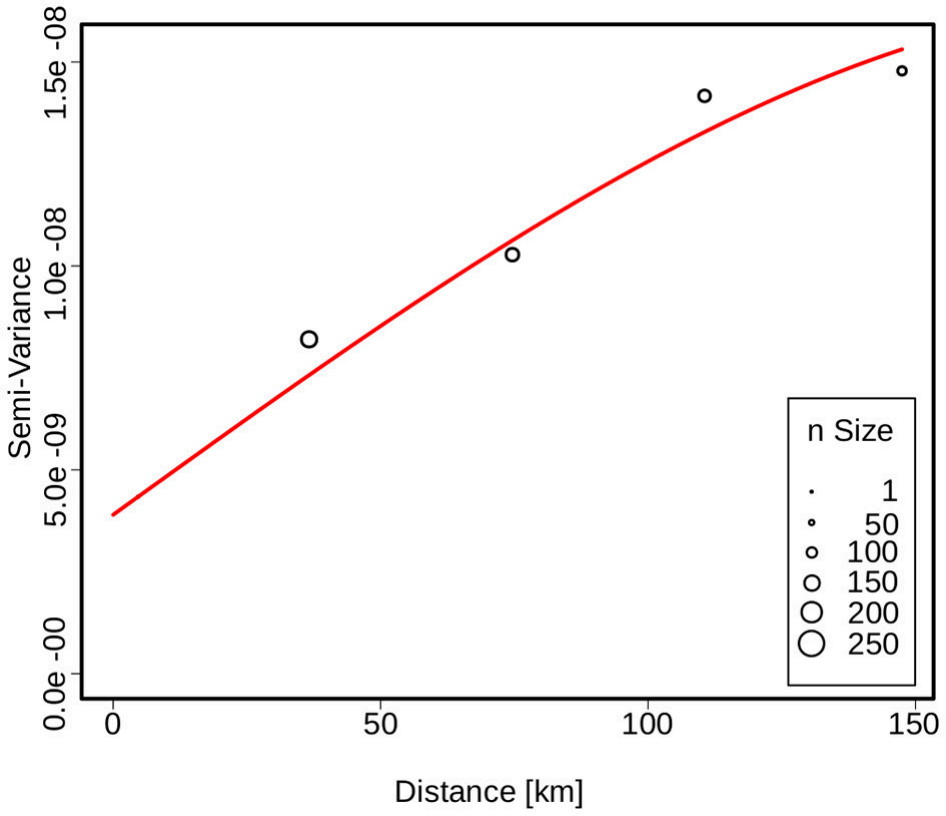
Semivariogram showing the spatial dependence of the genetic similarity between two strains as a function of distance. The size of the circles is proportional to the number of strains used for variance calculation. The red line indicates the fit of the spherical model used to describe the spatial structure.

**Figure 4:**
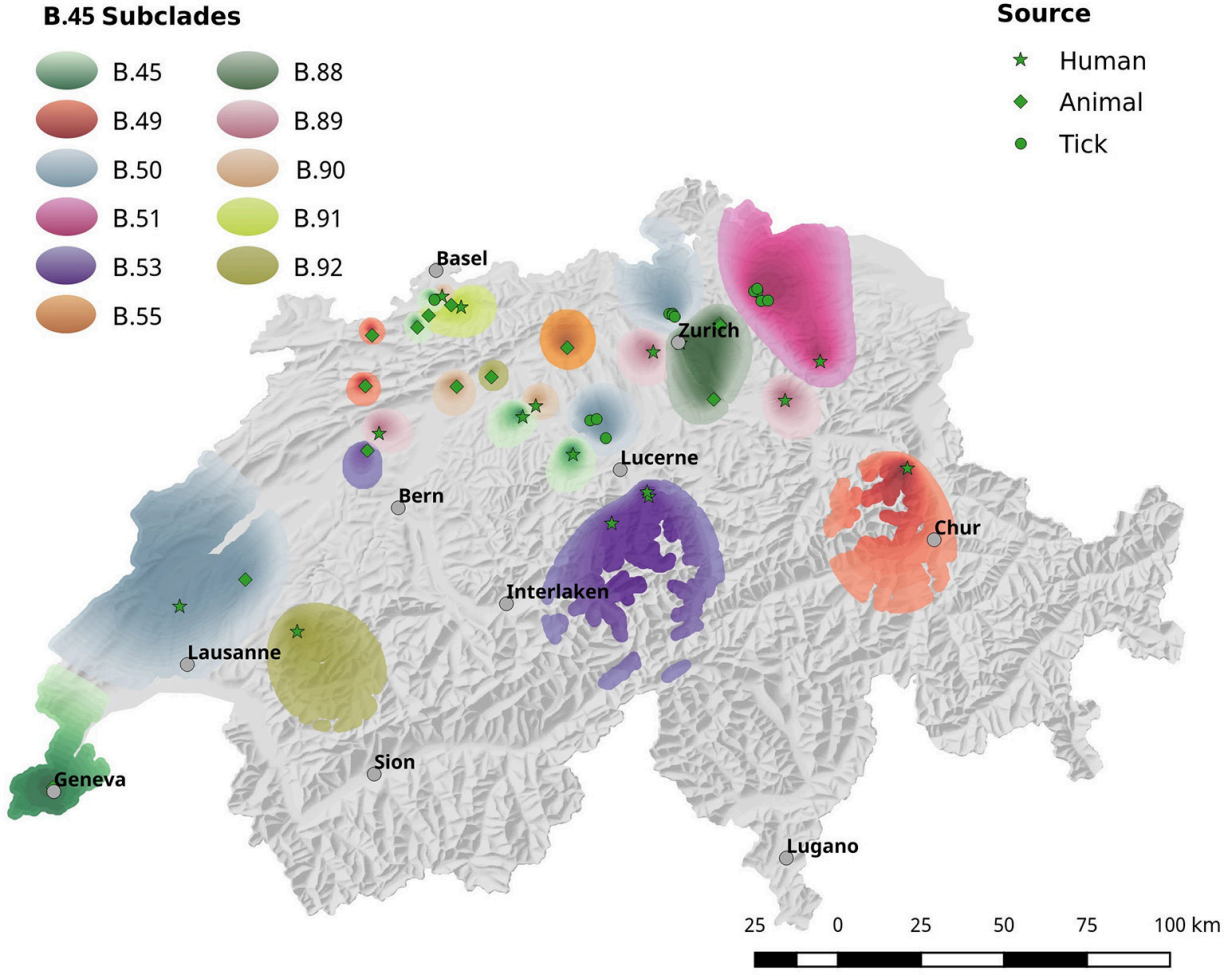
Spatial interpolation of phylogenetic clusters by krigin. Due to the high genetic diversity of *Fth*, the geographic distribution of the strains is shown in two separate maps. This Figure shows the interpolated distribution of the B.45 subclades. Figure 5 comprises the data for subclades B.33, B.86 and B.46.

**Figure 5:**
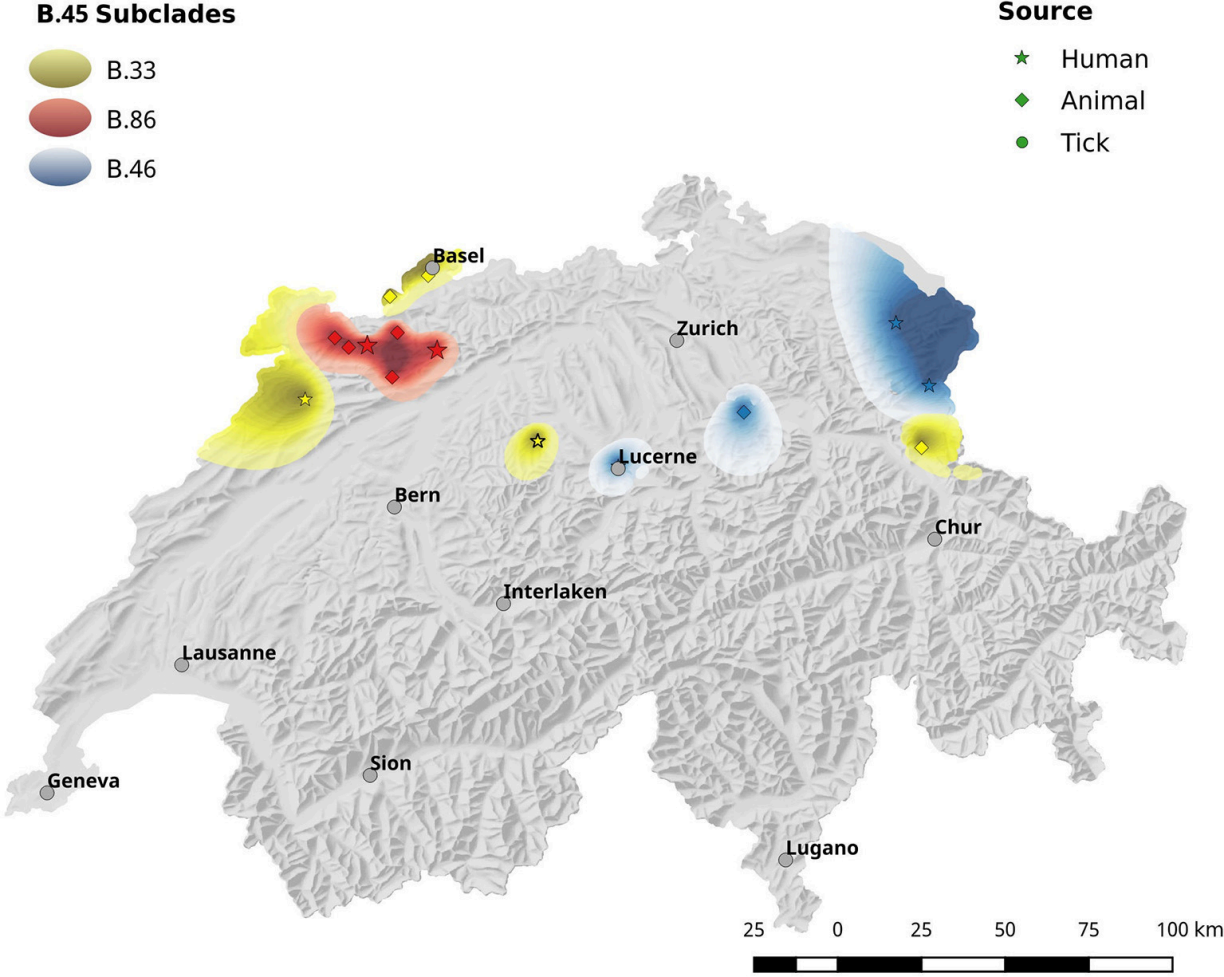
Same as Figure 4.

The spatial dependence of the genetic variation among the isolates is represented by the semi-variogram shown in Figure 3. The good fit (*R*^2^ = 0.96) of the applied spherical model clearly indicates a statistically significant dependence between genetic variation and geographic distance. The Kriging interpolation (Krige, 1951), based on the model derived from the semi-variogram, shows that clade B.45 is spread over the majority of the Swiss territory below 1,500 m of altitude. The subclades of B.45 that show a higher spatial coherence are visible as focal hotspots (Figure 4). The clades B.46 and B.86 show a geographically more focused distribution (Figure 5). Three animal and one human isolate assigned to the Northeast European B.12 clade originated from areas close to the northern and eastern Swiss border. One human isolate was isolated in central Switzerland.

### Genetic variation in the Swiss *Fth* population

*Breseq* analysis revealed 259 polymorphisms within the 59 sequenced *Fth* genomes relative to the genome FTNF002-00. We identified 36 deletions, 28 insertions and 195 SNPs. Seventy-nine SNPs were non-synonymous. Of the 116 synonymous SNPs, 46 were located in coding regions of the genome. Regarding the mutations that were exclusively found in the newly defined B.86 subclade, 9 of the in total 17 SNPs were non-synonymous. Two non-synonymous mutations were found in genes associated with the heme (*hemA*) and cytochrome c (*FTA_0224*) biosynthesis. One mutation alters a member (*FTA_0090*) of the major facilitator superfamily (MFS) which is involved in the transport of small molecule substrates across all classes of organisms and plays an important role in sustaining the osmotic balance between the cytosol and the environment. The remaining 5 non-synonymous SNPs are found in genes coding for proteins involved in the carbohydrate (*FTA_0510, FTA_1026, FTA_1126)* and fatty-acid (*FTA_0617*) metabolism of *Fth* (Table 3).

**Table 3:** List of the non-synonymous SNPs identified with the *breseq* pipeline.

**Position**	**Mutation**	**Annotation**	**Gene**	**Description**
78116	C → T	V40I (GTT → ATT)	*FTA_0090 ←*	Major facilitator family (MFS) transporter protein
207830	G → A	G61D (GGT → GAT)	*FTA_0224 →*	Hypothetical membrane protein/putative cytochrome c-type biogenesis protein
468941	C → T	T425I (ACT → ATT)	*FTA_0510 →*	Phosphoglucomutase/phosphomannomutase
563329	C → T	G221R (GGG → AGG)	*FTA_0617 ←*	Acetyl-CoA acetyltransferase
944751	G → T	S162I (AGT → ATT)	*FTA_1026 →*	Iron-containing alcohol dehydrogenase
1022282	G → T	R456M (AGG → ATG)	*FTA_1126 →*	Phosphorylase family 2/alpha-beta hydrolase fold protein
1022285	A → G	D457G (GAC → GGC)	*FTA_1126 →*	Phosphorylase family 2/alpha-beta hydrolase fold protein
1649613	A → C	I65R (ATA → AGA)	*hemA ←*	Glutamyl-tRNA reductase

*The genomic positions correspond to the FTNF002-00 strain (NC_009749.1)*.

All five strains assigned to the B.12 clade showed the SNPs in the *rrl* (23S rRNA) gene (A2059C and A453G) which were found to be responsible for the erythromycin resistance, which is characteristic for this clade (Karlsson et al., 2016). These findings are in agreement with the antibiotic susceptibility testing and MLVA typing results of Origgi et al. performed on the same isolates (Origgi et al., 2014).

All genetic variations found in the core genome alignment with the strain FTNF00-002 as reference are included in a variant call file (vcf) as Supplementary Material (Supplementary Table 1).

### Epidemiology of tularemia in Switzerland

**Figure 6:**
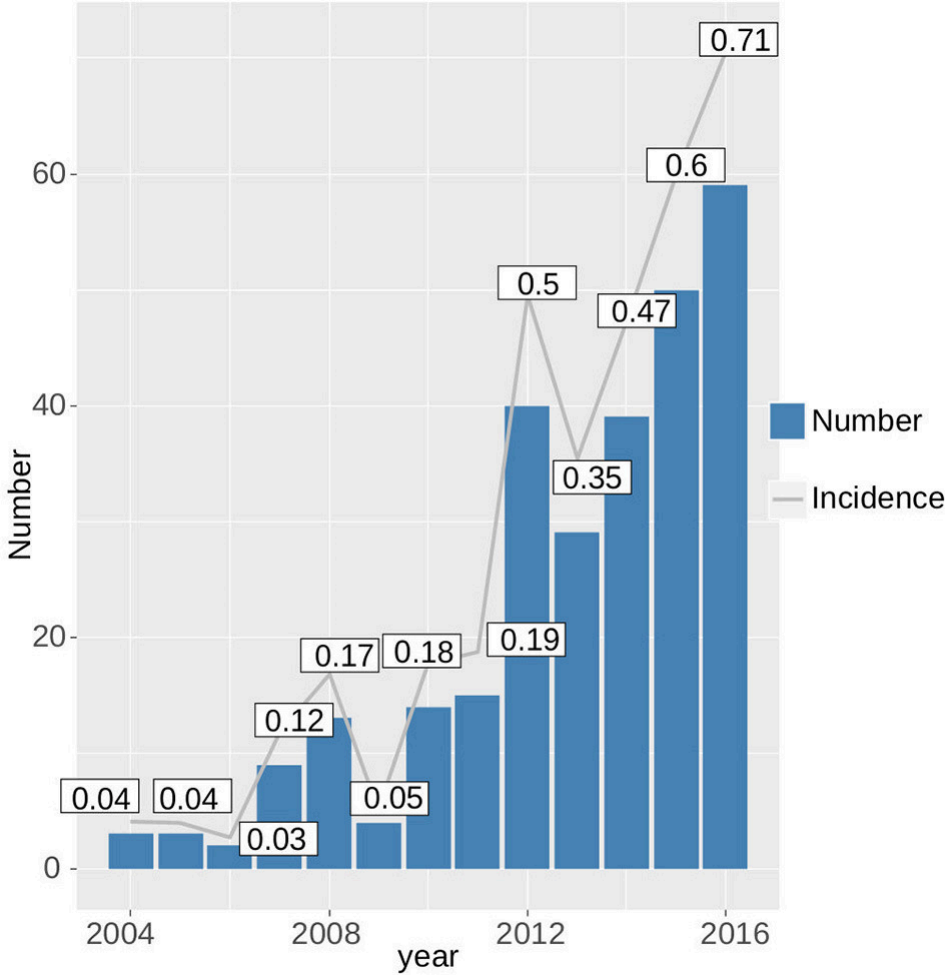
Increase in the absolute case numbers and incidence of tularemia in Switzerland from 2004 to 2016.

**Figure 7:**
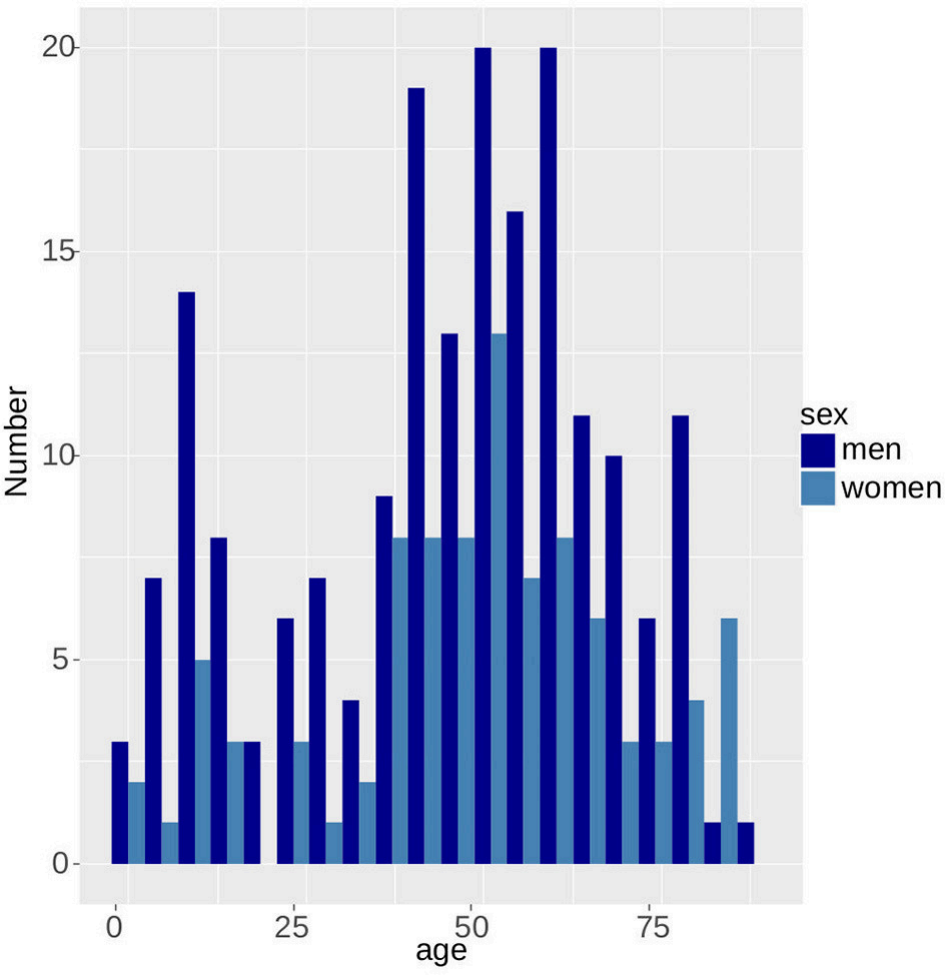
Bimodal age distribution of tularemia patients. Men are more likely to be affected than women.

Since 2004, the incidence of tularemia in Switzerland has risen ~15-fold from 0.04 to 0.69 per 100,000 per year (Figure 6). With the exception of patients >75 years of age, males were generally more affected by tularemia. We observe a bimodal distribution of case numbers per age group with a narrow peak between 10 and 12 years of age and a broader peak spanning the ages from 27 to 75 years (Figure 7).

In agreement with a terrestrial lifecycle, *Fth* isolates from humans, animals and ticks are represented in the same terminal subclades. In subclade B.51, the tick derived isolates FT40/FT47 show no differences in the 1,474,944 bases core genome alignment with the human FT75 isolate. The same applies for the isolates FT05 (human) and FT58 (animal) in clade B.90.

**Figure 8:**
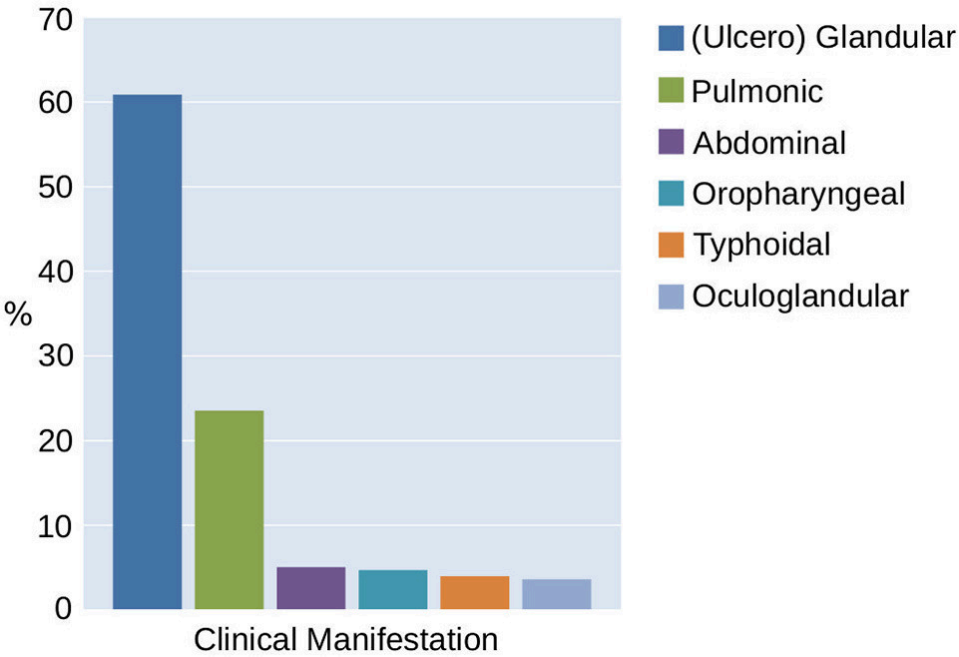
Classification of tularemia in Switzerland between 2004 and 2016 according to the clinical manifestation.

The majority of the 276 patients reported an insect or arthropod bite prior to the occurrence of tularemia symptoms (33% tick-bite, 24% insects—total 57%). Contact with wild animals was assumed as the cause of 22% of the infections. In 23% of the cases, the source was unknown. The proportion of the cases with a suspected vector-borne source of tularemia infection correlated with the clinical manifestation of the disease. The glandular/ulceroglandular type typically associated with the bite of a hematophagous arthropod was observed in 60% of the cases. The pulmonary type was represented in 24% and the abdominal/oropharyngeal type in 14% of the patients (Figure 8). Hence, 38% of the 276 reported infections can be attributed to direct or indirect contact with infected wild life.

**Figure 9:**
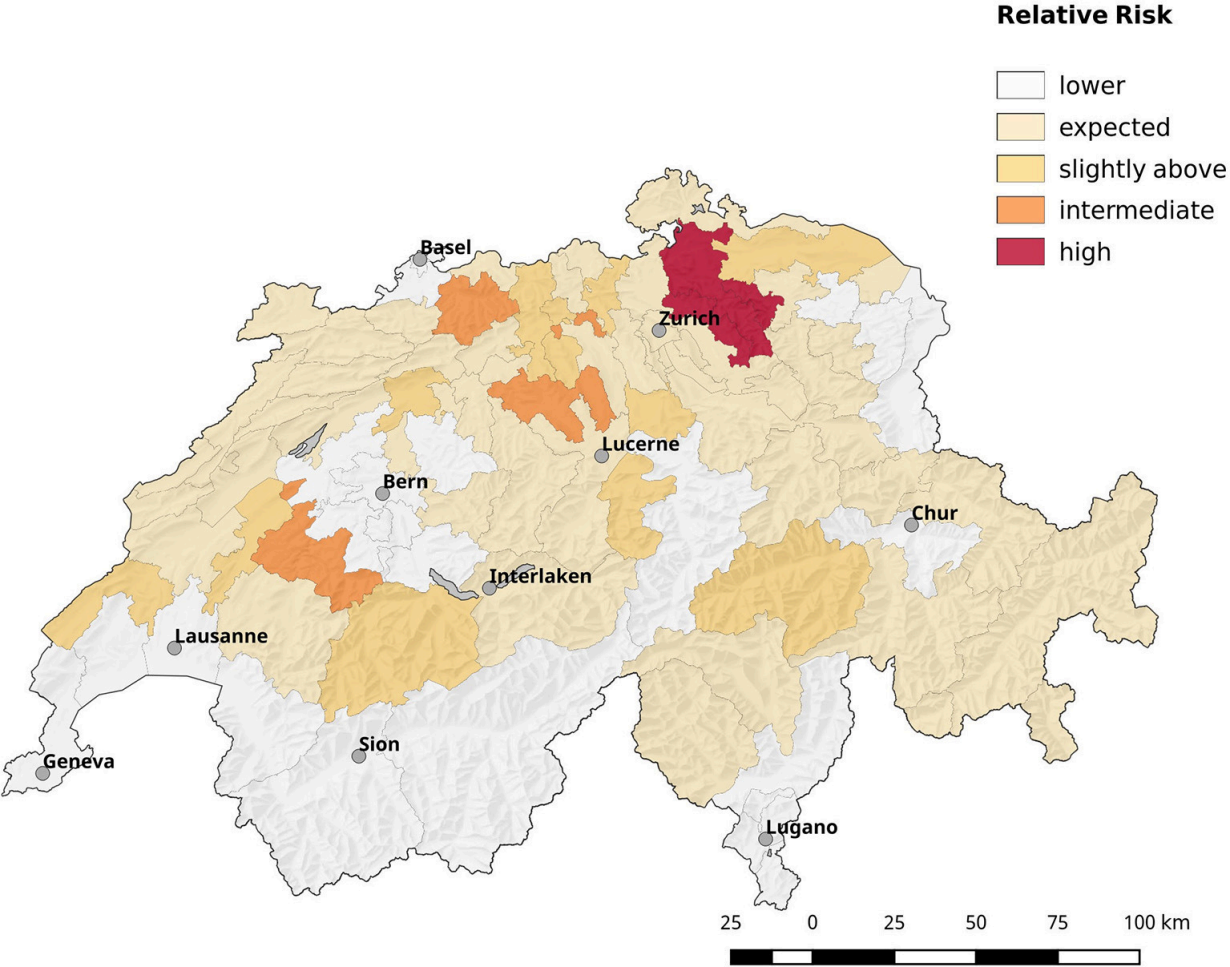
Spatial distribution of cases by postal code area (first two digits). The estimated relative risk is shown, which is defined by the ratio of observed cases to expected ones. Most areas have less cases than expected, e.g., the relative risk is below 1 (blank zone). The remaining areas are shaded from orange to red according to their relative risk value.

**Figure 10:**
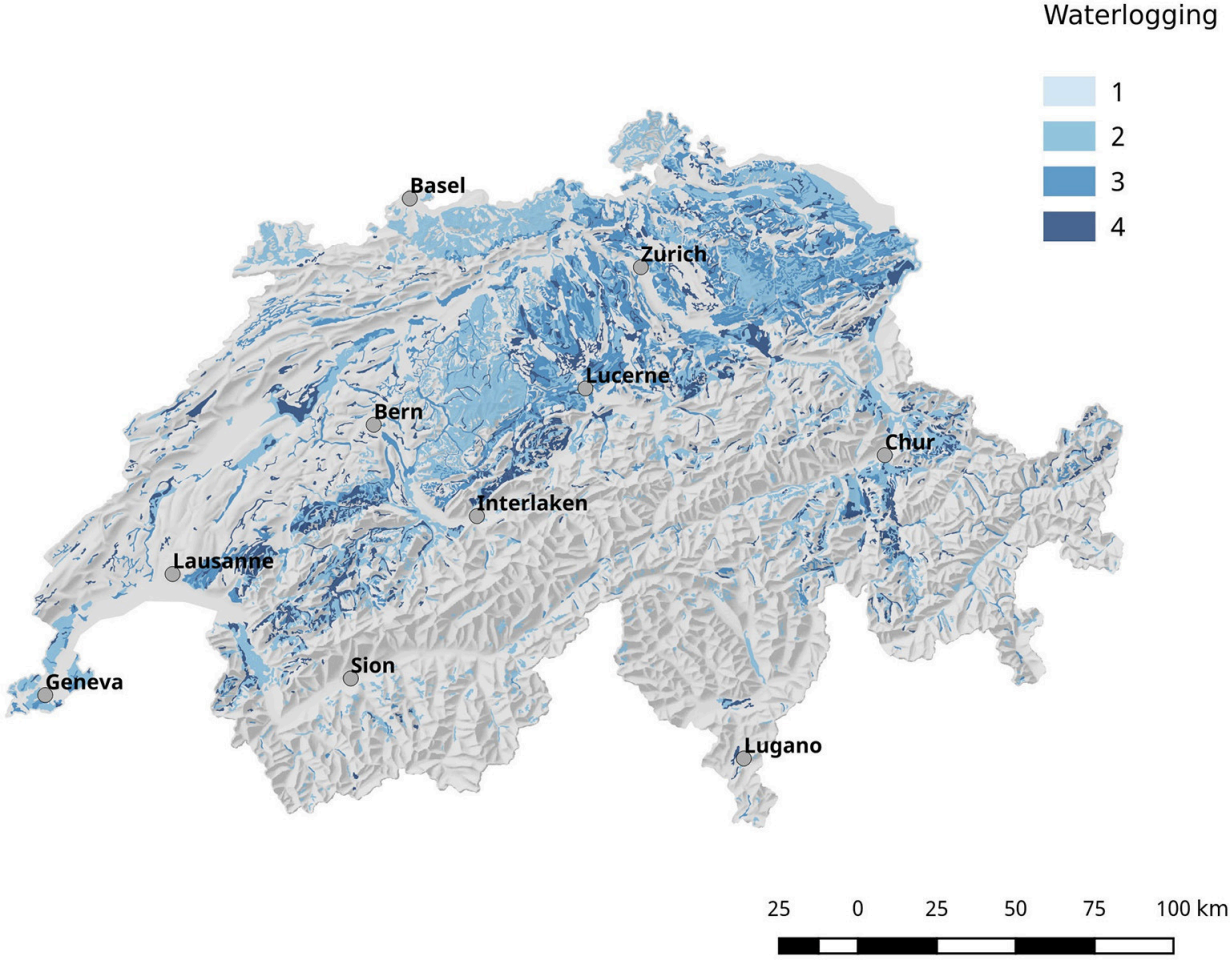
Geographical overview of the water saturation of the soil (waterlogging). In general, the soil in the north-eastern part of Switzerland shows a higher degree of water saturation compared to other regions.

**Figure 11:**
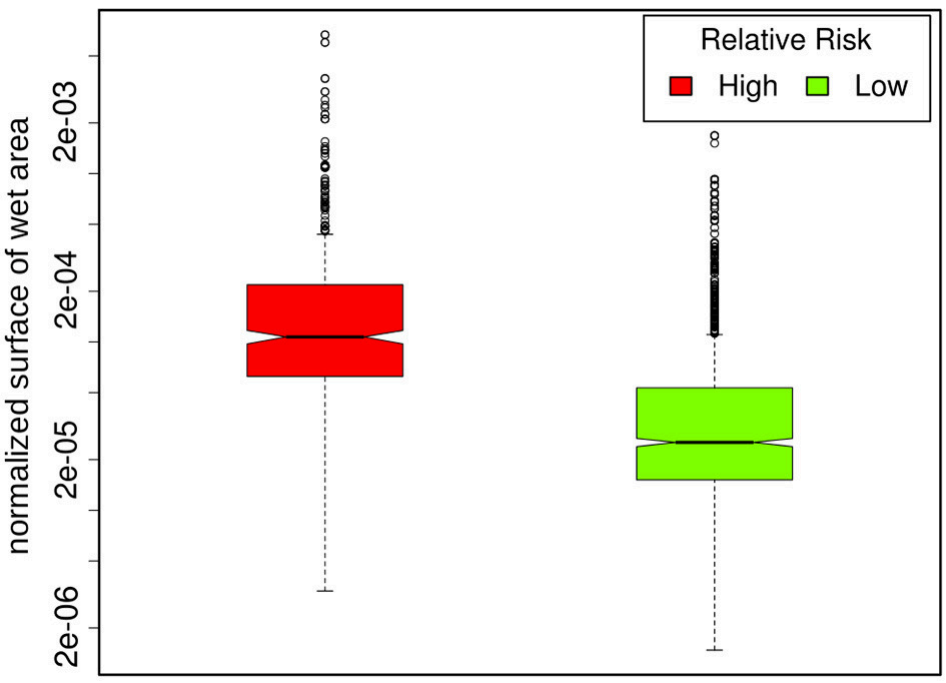
Regions with an elevated relative risk of tularemia show significantly higher degrees of waterlogging compared to low risk regions (*p* = 1.2 ^*^ 10^−15^).

Population corrected risk estimation on the spatial distribution of tularemia cases shows an uneven distribution. Areas with elevated relative risk are predominately located in the northeastern area of Switzerland (Figure 9). Our data suggest that ticks are the predominant source of infection in Switzerland. Therefore, we correlated the risk estimations with climatic as well as ecological factors known to influence the survival and vector competence of ticks. Relative humidity (RH) and saturation deficit (SD) are known to be of pivotal importance for tick abundance and survival. Consequently, we compared the surface normalized averages of waterlogging levels in areas with elevated risk for acquiring tularemia vs. low risk areas and found a highly significant effect of soil moisture (Figures 10, 11).

**Figure 12:**
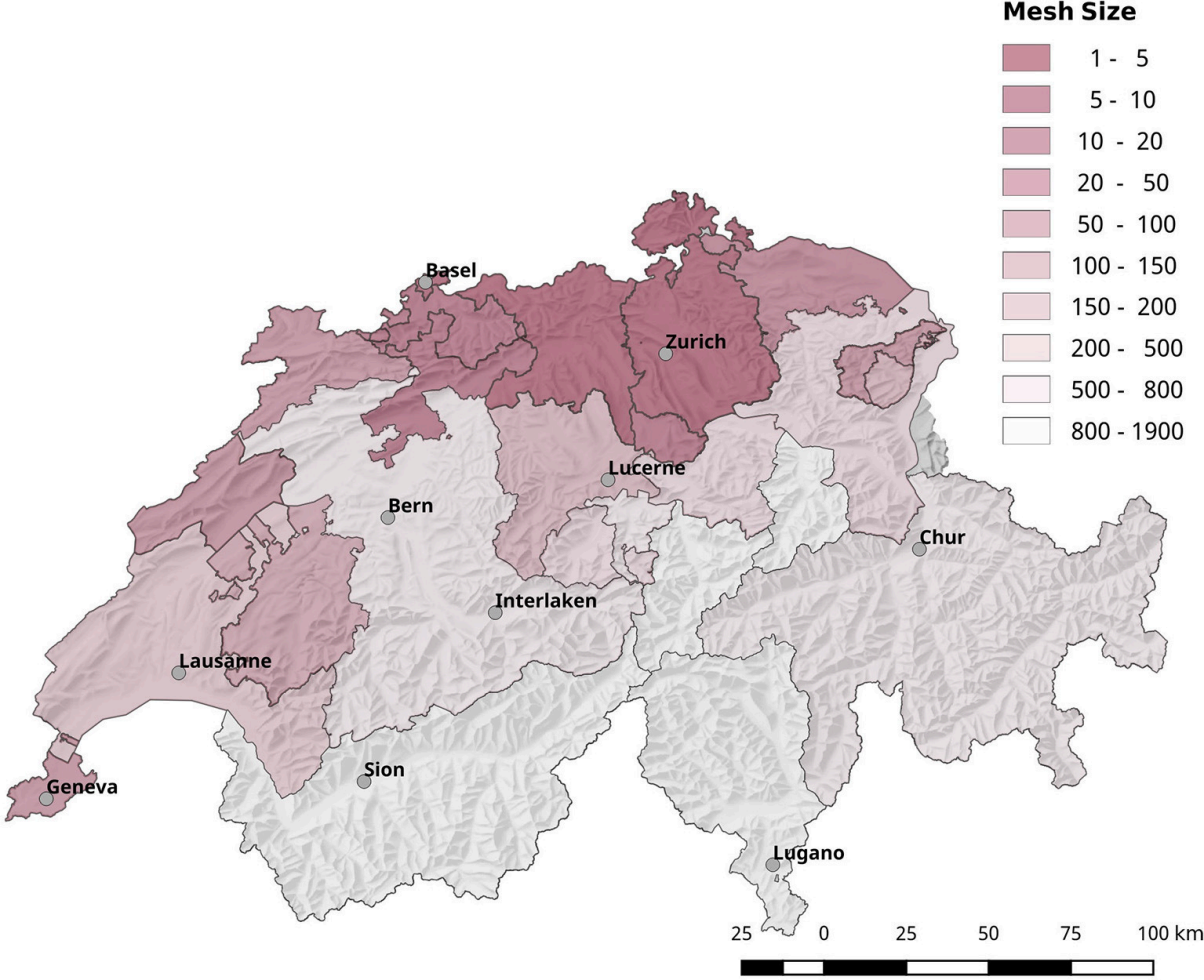
Geographical overview of the degree of landscape fragmentation of the Swiss cantons. Most of the regions with a high risk for acquiring tularemia are also highly fragmented.

The reduction of biodiversity due to urbanization influences the competence of vectors by shifting the ratio of competent to non-competent hosts. Most of the regions with an elevated risk for acquiring tularemia are highly fragmented and show below average mesh sizes (Figure 12).

## Discussion

### Prevalence, genetic diversity, and population genomics of Swiss Fth isolates

The determined prevalence of 0.2‰ is low compared to other pathogens found in ticks (Oechslin et al., 2017) and confirms principally a previous smaller survey of the Swiss Army (Wicki et al., 2000).

The successful cultivation of *Fth* from ticks substantiate the role of *I. ricinus* as important vector of the pathogen. Furthermore, the availability of isolates was a prerequisite for the WGS inferred high-resolution phylogeny.

As described elsewhere (Origgi et al., 2014) the B.13 and B.11 strain are co-circulating in Switzerland with a predominance of the latter. Strikingly, the geographic origin of four of the five B.13 isolates is close to the northern and eastern Swiss border. Since the B.11 and B.13 strains are equally established in the German hare population (Müller et al., 2013) and B.13 prevails in Austria (Tomaso et al., 2005), our data suggests a sporadic intrusion of the B.13 strain from neighboring countries.

In accordance with recent publications regarding the dispersal of *Fth* in Europe (Dwibedi et al., 2016), Switzerland seems to harbor an exceptionally high genetic diversity of the pathogen compared to other European countries. The phylogenetic structure and proportion of the B.11 subclades derived from Swiss isolates are almost identical to the one reported in the European survey. In this aspect, Switzerland can be considered as a small-scale model for *Fth* population structure. Moreover, the identification of six additional B.11 subclades underlines the notion that the alpine region harbors an evolutionary older and highly diverse founder population (Dwibedi et al., 2016).

A major factor that is positively associated with the biodiversity of an ecosystem is the topographic complexity of a habitat (Zhou et al., 2015). Together with climate-related factors, topography is known to influence a wide array of environmental parameters including hydrology, nutrient dispersion, soil structure, and the microclimate. Furthermore, mountain ranges on the scale of the Swiss Alps form a relevant barrier for animal migration. In this light, the observed diversity of the B.11 clade in Switzerland and the statistically significant correlation between genetic- and geographic distance may reflect small-scale topographic fragmentation of the habitat of the susceptible hosts and pathogen vectors.

### Analysis of a newly identified B.11 subclade

In agreement with the European survey, the B.45 subclade of B.11 was found to be the most successful subclade in Switzerland in terms of geographical distribution and prevalence. Noticeably, all of the 14 tick isolates from five different geographic regions were assigned to the B.45 clade, whereas B.46 and B.86 were solely comprised by human and animal isolates. One possible explanation for this observation could be that the strains of the B.45 subclade are better adapted to the arthropod vector and thus elevate the vector competence of the ticks, compared to ticks carrying B.46/B.86 strains. A hint for this line of argumentation is provided by our *breseq* data. Seven non-synonymous SNPs were found exclusively in the B.86 subclade of B.11, which might modulate the function of proteins involved in intracellular survival. Members of the MFS are involved in the transport of small molecule substrates across all classes of organisms and play an important role in sustaining the osmotic balance between the cytosol and the environment. In *F. tularensis*, phagosomal transport proteins that are a sub-family of MFS were identified as factors essential for lethality to adult fruit flies (Akimana and Kwaik, 2011). Furthermore, members of the MFS class are discussed as a target for attenuation and vaccine development (Marohn et al., 2012). The tolerance to oxidative stress and the acquisition of iron are fundamental aspects for the survival of a pathogen in a host environment. Besides its pivotal role in cellular respiration, the iron containing porphyrin-ring heme is involved in the function of a variety of enzymes like catalases and nitric oxide synthase (Choby and Skaar, 2016). In this light, the non-synonymous mutation of hemA that is involved in the first steps of heme biosynthesis may affect the tolerance of clade B.86 strains to oxidative stress (Ezraty et al., 2017). Additionally, we identified a mutation in another component associated with the electron transport chain: cytochrome c-type biogenesis proteins are membrane-bound proteins that may play a role in the guidance of apocytochromes and heme groups for their covalent linkage by the cytochrome-c-heme lyase. In summary, the mutations of proteins involved in the adaption to environmental conditions may modify the fitness of the B.86 subclade and may prevent persistence in ticks. This would lead to a diminished host range and thus restricted geographical dispersal. However, this interpretation should be treated with caution and needs to be substantiated further by functional studies.

### Investigation of eco-epidemiological aspects

According to the reported tularemia cases, ticks are the predominant vector for disease transmission in Switzerland over the last 10 years. The importance of arthropod vectors is also reflected in the clinical manifestation of the disease where the glandular / ulcero glandular form prevails. These findings together with the sporadic occurrence and the low number of cases are in agreement with a terrestrial life cycle of *Fth* involving rodents, lagomorphs and ticks as main source for human infections. Despite the lacking evidence of a transovarial transmission of *Fth* in *Ixodes ricinus* (Genchi et al., 2015), it is known that the pathogen is propagated transracial from larvae to nymphs and adults in the 3-year life cycle of the vector. Therefore it was suggested that ticks can be regarded as a *de facto* reservoir of tularemia (Petersen et al., 2009; Foley and Nieto, 2010).

Our spatial data from tick, human, and vertebrate isolates, as well as from reported clinical cases suggest that the *Fth* population is not randomly distributed in Switzerland. The highest risk for acquiring tularemia coincides with regions where *Fth* was found in the ticks, a hint to the importance of their role as a biological niche for *Fth*. Since the initial tick survey was conducted with the aim to assess the prevalence of TBEV in all regions of Switzerland below 1,500 m altitude (Gäumann et al., 2010), the identification of *Fth* foci within the TBEV sample pool was not biased by the number of reported tularemia cases in a given region.

### Analysis of habitat characteristics and abiotic factors influencing the tick abundance

An important factor influencing the distribution of the pathogen in Switzerland is the complex topography of the country where 50% of the territory lays above an altitude of 1,000 m above sea level. Only one case of tularemia was reported in a patient living at 1,700 m altitude and contaminated water or food was suspected to be the source of infection (Ernst et al., 2015). In principle, the incidence of tularemia shows the same altitude dependence as was shown for borreliosis, where the prevalence of the pathogen in tick nymphs correlated negatively with altitude (Gern et al., 2008). There are multitudes of additional environmental parameters that are known to have an influence on the transmission dynamics of tick-borne pathogens. Abiotic factors like temperature, wind, RH and SD are recognized to be of pivotal importance for tick abundance and survival (Vial, 2009; Alonso-Carné et al., 2015). It was shown that a RH of 85% is required for the survival of *Ixodes ricinus* in its non-parasitic stage (Keymer et al., 2009). Studies, which assessed the usability of satellite-based remote sensing data as indices for geospatial disease monitoring, identified soil moisture as an important factor for the establishment of tick habitats (Beck et al., 2000; Barrios et al., 2012).

**Figure 13:**
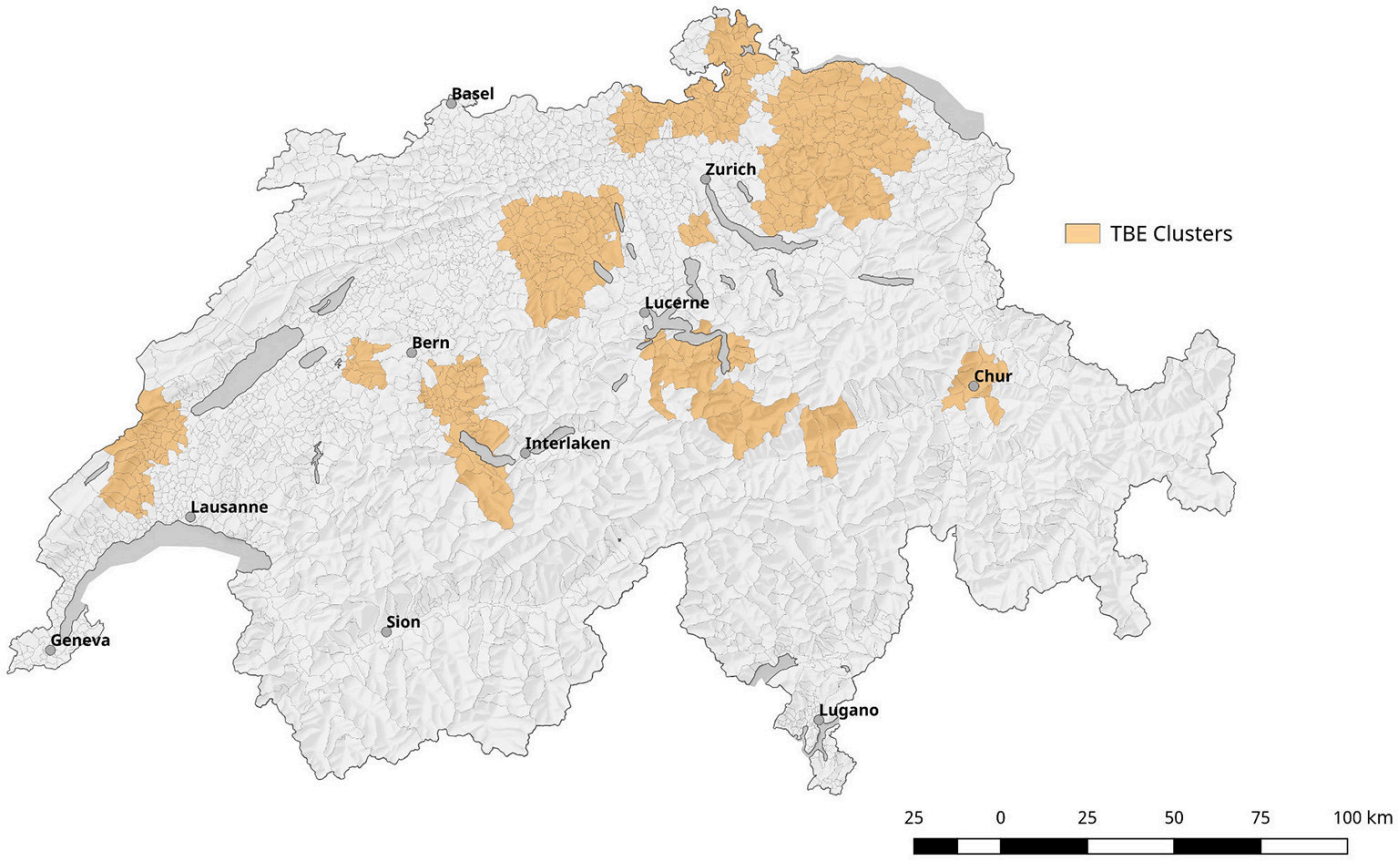
Depiction of localized clusters of Tick borne encephalitis (TBE). It is based on information from the mandatory reporting system between 2007 and 2016.

Comparing surface normalized averages of waterlogging levels in areas with elevated risk for acquiring tularemia vs. low-/expected risk areas, we find a highly significant effect of soil moisture (Figures 10, 11). Thus, the elevated prevalence of *Fth* in ticks and the higher incidence of tularemia in the northeastern part of Switzerland may at least be partially explained by the above average soil moisture in this region, favoring tick survival during dry periods. Another observation that fits in this line of argumentation is the congruence of high-risk tularemia regions with areas of elevated risk of Tick Born Encephalitis (TBE) (Figure 13).

### Habitat fragmentation and urbanization in Switzerland

Besides micro-climatic factors, anthropogenic changes of the ecosystem, and social factors are known to have a fundamental impact on the host-vector-pathogen dynamics (Bayles et al., 2013; Lou et al., 2014). As seen in other European countries, the fragmentation and urbanization of the Swiss landscape is on the rise which is reflected in a linear decrease of the “effective mesh size” during the last 70 years. The mesh size is a metric that describes the probability that two random points in the landscape can be connected without the interference of artificial structures, for example, transportation routes, buildings or developed land (Jaeger, 2000, 2006). The more barriers fragmenting the landscape, the lower the probability that two points are connected, and the lower the effective mesh size. In an ecological view, the mesh size can be interpreted as the probability that two animals of the same specie find each other in the landscape. Therefore, the reduction of the mesh size due to progressing landscape fragmentation may reach a limit where a given species is no longer able to sustain a stable population, which reduces the biodiversity of the area (Di Giulio et al., 2009). Most of the regions with an elevated risk for acquiring tularemia are highly fragmented and show a below average mesh sizes (Figure 12). The reduction of biodiversity may alter the ratio of hosts vs. low or non-competent hosts, thereby reducing the pathogen dilution effect. A diminished pathogen dilution effect may in turn elevate the vector competence of the ticks and thus the probability of disease transmission (Schmidt and Ostfeld, 2001; Pfäffle et al., 2013; Dantas-Torres, 2015).

### Risk factors for human disease

The bimodal distribution of the tularemia cases per age group and the predominance of male patients is an indicator of social factors influencing the transmission probability of the disease and is in accordance with the incidence in other countries (Desvars et al., 2015). According to clinicians treating patients in the elevated risk areas, the peak in the age group of the 8–12 year old children is due to the joining of youth organizations like the scouts that are combing through the forests away from fixed paths seeking for adventure. The second peak with a maximum at 50 years reflects the demographic age structure of the Swiss population.

## Conclusion

The combination of high-resolution whole genome phylogenies with epidemiological and ecological parameters allows a profound insight into the transmission modalities of tularemia. The exceptionally high genetic diversity of the Swiss *Fth* population allows us to study the population and disease dynamics of the pathogen in a small geographical context. In our work, we found evidence for the spatial correlation of disease incidence and ecological/anthropogenic factors that influence the survival and vector competence of the local tick population.

Based on these findings, we conclude that *I. ricinus* plays a pivotal role in the establishment of the disease in a given ecological niche and in sustaining the transmission cycle.

## Author contributions

MW analyzed the genomic data, performed the spatial statistics, and drafted the manuscript. EA compiled the epidemiological data and calculated the spatial relative disease risk. PP provided the animal isolates and expertise in the field of tularemia. SG performed the functional SNP analysis and provided the expertise in whole genome phylogeny. CB, RA-G designed and organized the tick sampling and provided expertise in tick borne diseases. UK provided the expertise in the clinical aspects of tularemia. DJ, RG gave theoretical and practical advice for bacterial isolation and provided expertise in the eco-epidemiology of tularemia. NS coordinated and supervised the presented work. All authors contributed to manuscript revision, read, and approved the submitted version.

### Conflict of interest statement

The authors declare that the research was conducted in the absence of any commercial or financial relationships that could be construed as a potential conflict of interest.

## Supplementary material

Supplementary Table 1 Genetic variations found in the core genome alignment with the strain FTNF00–002.
